# l-Menthol attenuates the magnitude of cold-induced vasodilation on the extremities of young females

**DOI:** 10.1186/s40101-018-0174-x

**Published:** 2018-05-09

**Authors:** Siyeon Kim, Joo-Young Lee

**Affiliations:** 1 COM:FORT Laboratory, College of Human Ecology, Seoul National University, 1 Gwanak-ro, Gwanak-gu, Seoul, 151-742 South Korea; 2 Research Institute of Human Ecology, Seoul National University, Seoul, Korea

**Keywords:** Cold-induced vasodilation, Arteriovenous anastomoses, Menthol, Vasoconstriction, Cold-pain sensation

## Abstract

**Background:**

Menthol chemically triggers cold-sensitive receptors in the skin without conductive skin cooling. We investigated the effects of menthol-induced activation of cutaneous cold receptors on the cold-induced vasodilation (CIVD) of the finger. We hypothesized that the menthol application would attenuate typical CIVD responses.

**Methods:**

1.5% l-menthol was fully applied over the left hand and forearm, and then, the middle finger of the left hand was immersed into 4 °C water for 30 min. A trial consisted of 10-min rest followed by 30-min immersion and 20-min recovery in 28 °C air temperature with 20% relative humidity. Another trial without the menthol application was carried out as a control. Seventeen females (24.2 ± 2.6 years in age, 160.5 ± 5.1 cm in height, and 51.2 ± 5.7 kg in body weight) participated in the two trials.

**Results:**

The results showed that the maximum and average temperatures of the finger during the water immersion were lower in the menthol application when compared to control (*P* < 0.05), whereas no significant differences appeared in the onset time of CIVD, the frequency of CIVD, and minimum finger temperature. These results imply that stronger stimulation of cold receptors without additional conductive skin cooling did not attenuate the triggering of CIVD responses but intensified vasoconstriction after the first occurrence of CIVD.

**Conclusion:**

It is suggested that substantial and conductive heat loss through the skin along with activation of cold receptors may be required to retain rewarming at a certain level.

## Background

Cold-induced vasodilation (CIVD) is an acute increase in local blood flow during cold exposure on the glabrous skin. Because decreases in skin temperature during extreme cold exposure can result in cold injuries, CIVD is a physiologically beneficial phenomenon that prevents localized damage against freezing, and facilitates adequate dexterity and tactile sensitivity during work in cold environment [1, 2]. Arterio-venous anastomoses (AVAs) are considered to play a primary role in the occurrence of CIVD [3], but the underlying mechanisms have not been completely explored. AVAs are under the influence of the sympathetic adrenergic vasoconstrictor nerves, and the reduction of cutaneous vasoconstrictor activity could result in the opening of AVAs. This process has been supported by the role of norepinephrine (NE) released from adrenergic nerve endings [4]. Marked CIVD with core temperature increased by 0.5 °C or high body heat content supported the thermoregulatory role of CIVD contributing heat loss [5, 6].

Along with the centrally driven mechanism, there could be other locally driving mechanisms including the direct influence of cold on vascular smooth muscle or the release of vasodilating substances, such as nitric oxide [1, 5, 7, 8]. Sendowski and his colleagues [8] compared the following three conditions to examine sympathetic stimulation during cold water immersion: right index finger immersion (T1), right hand immersion (T2), and simultaneous right index finger and left hand immersion (T3). They found that T1 and T3 did not show significant differences in onset time of CIVD, but T2 onset time was significantly slower than T1. These results implied that the enhancement of cooling on the adjacent skin could attenuate triggering CIVD. Furthermore, several other previous studies claim that CIVD responses cannot be always explained by one mechanism only [9, 10]. Among several methodologies for segmenting the CIVD mechanism, differentiating the cooling area could be a useful manipulation for investigating the contribution of the sympathetic nervous system [8]. Nevertheless, we cannot fully exclude the possibility of the influences of conductive heat loss induced by direct skin cooling on blood/tissue temperature or on the intensification of the sympathetic adrenergic vasoconstrictor tone. This implies that another approach which facilitates direct stimulation of cold receptors without direct skin cooling could contribute to separating such factors and could be implemented by using a chemical stimulator, l-menthol.

Menthol is a naturally or chemically synthesized compound that induces coolness on the skin and oral cavity. The role of TRP melastatin-8 (TRPM8) has been acknowledged as a theroreceptor on which l-menthol activates [11]. TRPM8 is activated both by moderate cold at a temperature below ~ 25 °C [12] and by cooling agents such as l-menthol. l-Menthol virtually shifts thermal thresholds toward a higher temperature [13], which results in the sensitization of cool and warm sensations. More recently, with the development of an antagonist of TRPM8, it has been revealed that TRPM8 is a universal cold receptor in the thermoregulation system which could affect several cold-defense responses: cold-avoidance behavior, skin vasoconstriction, and nonshivering thermogenesis in brown adipose tissue [14].

In this study, l-menthol was utilized to activate cutaneous cold receptors with minimal skin cooling of the adjacent skin during a finger immersion test. We hypothesized that the application of l-menthol on the hand and forearm would attenuate the CIVD responses of the finger, which would prove the influence of the sympathetic nervous system with minimum conductive skin cooling. Secondly, we hypothesized that the onset time of CIVD in the finger would not be influenced by the menthol application, which was based on Sendowski et al. [8] reporting that the CIVD onset time was influenced by just local skin cooling not by systemic sympathetic activity.

## Methods

### Subjects

Seventeen Korean female students [mean ± SD, 24.2 ± 2.6 years in age, 160.5 ± 5.1 cm in height, 51.2 ± 5.7 kg in body weight] participated in the present study. All subjects were free of known cardiovascular dysfunction and fully informed of the purpose and potential risks of the present study. The menstrual cycle was not considered in this experiment because in previous studies, the vasoconstrictor response to local cooling was not influenced by reproductive hormone status [15]. The present study was approved by the Institutional Review Board of Seoul National University (IRB No.1504/002-003).

### Experimental procedures

All subjects participated in two local cold tests: menthol application and water application (control). To avoid the order effects, the experimental orders of the two trials for each participant were randomized and the two trials were separated by at least 2 days within 2 weeks. All experiments were conducted in November, which is Autumn in South Korea. To avoid any internal thermoregulatory differences caused by a circadian rhythm, each subject was asked to visit the laboratory in an identical time in the afternoon for their own trials. All participants refrained from alcohol for the previous 24 h, along with refraining from any food and caffeine for 3 h prior to their scheduled tests.

A 1.5% l-menthol solution was prepared by mixing 44.5 g of 35.23% menthol oil in 1 l of water. Each subject completely soaked their left hand and forearm in the menthol solution. The average amount of applied menthol solution for a subject was 3.9 ± 1.6 g per trial. Subjects in the control group did not soak their hand and forearm. Experimental trials were not blinded to subjects. The skin where menthol solution was applied was totally dried up before the commencement of experimental trial so that only chemical stimulation on cold receptors was employed. A CIVD test commenced 20 min after the application of menthol solution. Menthol solution dried from the skin of the subjects during the 20 min. The experimental protocol of the local cold test consisted of 10-min stabilization followed by 30-min immersion and 20-min recovery. For the 30-min immersion, subjects immersed the middle finger of the left hand (by the proximal phalange) into the cold water. Water was automatically circulated and maintained at 4 °C by a refrigerated bath circulation (RW-0525G, JEIO TECH, South Korea), which was followed by the 20-min recovery where subjects kept their finger in the air at the level of the chest for 20 min. Subjects wore short sleeved t-shirts, long pants, socks, and their underwear (100% cotton), and total clothing insulation was estimated at 0.4~0.5 clo. Subjects sat on a chair in a climate chamber which was maintained at an air temperature of 28 °C with relative humidity of 20%RH during the whole experiment so that most subjects felt thermally comfortable at rest.

### Measurements and calculations

Skin temperatures were measured on the forehead, chest, anterior thigh, lateral calf, dorsal foot, forearm, dorsal hand, and volar side of the distal phalanx of the middle finger every 5 s using a data logger (LT-8A; Gram Corporation, Japan). Mean skin temperature was calculated according to Hardy and Dubois’ formula [16]. The parameters of the finger CIVD test were determined in accordance with those in Daanen [1]: minimum finger temperature (*T*_min_), time to initial increase in finger temperature from beginning of cold water immersion (*Onset time*), maximum finger temperature reached during cold water immersion (*T*_max_), time until appearance of *T*_max_ (*D*_*max*_), difference between *T*_max_ and *T*_min_ (Δ*T*), average finger temperature during the immersion after *T*_min_ (*T*_mean_), and the number of CIVD appearance (*Frequency*). *Frequency* was defined as an increase in the finger temperature > 2 °C after a vasoconstriction with a decrease in the finger temperature > 1 °C. Systolic and diastolic blood pressure (SBP and DBP, respectively) were measured two times every 5 min from the resting arm, and the averaged value was used for calculating mean arterial pressure (MAP). MAP was calculated by the following equation: MAP *=* DBP *+* (SBP − DBP)/3. Finger thermal sensations were rated every 5 min using the following categorical scale marked with nine numbers and corresponding verbal descriptors according to ISO 10551 (1995): − 4 (very cold), − 3 (cold), − 2 (cool), − 1 (slightly cool), 0 (neutral), 1 (slightly warm), 2 (warm), 3 (hot), and 4 (very hot). Finger cold pain sensation was rated every 5 min using a categorical scale marked with seven numbers with four verbal descriptors (modified from Strong and van Griensven [17]): 0 (no pain); 1, 2 (slightly painful); 3, 4 (painful); and 5, 6 (very painful). We did not measure core body temperature but started the finger immersion when they expressed themselves as being thermally comfortable after a 1~2-h rest at the climate chamber (28 °C and 20%RH).

#### Data analyses

A paired sample *t* tests were performed to compare two groups (menthol and control) regarding following variables: CIVD parameters (*T*_min_, onset time, *T*_max_, D_max_, Δ*T*, *T*_mean_, and frequency), and the changes between skin temperature at rest (*T*_rest_) and during recovery (*T*_rec_). In order to analyze the differences by phases, *T*_rest_ and *T*_immersion_ were expressed as the averaged values for the last 5 min during the 10-min rest and 30-min finger immersion, respectively, while *T*_rec_ was calculated by the averaged values for the last 3 min. To discern the significant differences between the two conditions by phases, two-way repeated measures analysis of variance (ANOVA) was used with the phasal factor (levels: rest, immersion, recovery) and the experimental conditions (menthol and control) with regard to the following independent variables: the middle finger, hand, forearm, and mean skin temperature, thermal sensation, and pain sensation. Two-way repeated measures ANOVA was also used to compare aforementioned skin temperatures at each minute to specify the time during CIVD using following factors: time (every minute) and conditions (levels: menthol and control). A significance was set at *P* < 0.05. Pair-wise comparisons were employed using paired sample *t* tests. All statistical analyses were performed with IBM SPSS Statistics 21.0. Values were expressed as means ± SD.

## Results

CIVD parameters on the finger

Among CIVD parameters, both *T*_max_ and *T*_mean_ showed significant differences between the two conditions (*P* < 0.05; Table 1). *T*_max_ was about 1 °C lower in the menthol condition (9.48 ± 2.27 °C) than that in the control condition (8.44 ± 2.02 °C) (*P* = 0.041). Likewise, *T*_mean_ was about 0.8 °C lower in the menthol condition (6.57 ± 1.29 °C) than that in the control condition (7.33 ± 1.51 °C) (*P* = 0.022). Though the onset time was slightly delayed in the menthol condition than that in the control condition, the difference between the two conditions was not significant (*P* = 0.563). The frequencies of CIVD for the 30-min immersion were on average two times in both conditions with no significant difference. *T*_min_ showed no statistical differences between the two conditions. One participant in the control condition did not show CIVD responses.

**Table 1:** Variables to characterize cold-induced vasodilation in a controlled condition and an experimental condition (menthol application)

	Control (CON)		Menthol		*P* value
	Mean	SD	Mean	SD	
*T*_min_ (°C)	4.20	0.77	3.88	0.17	N.S.
Onset time (min)	4.74	0.94	4.88	1.04	N.S.
*T*_max_ (°C)	9.48	2.27	8.44	2.02	0.041
*D*_max_ (min)	16.82	5.11	18.75	6.58	N.S.
Δ*T* (°C)	5.28	2.24	4.56	1.99	N.S.
*T*_mean_ (°C)	7.33	1.51	6.57	1.29	0.022
Frequency (times)	1.82	1.13	1.88	1.36	N.S.

*N* = 17 females

*T*_*min*_ minimum temperature in initial vasoconstriction, *Onset time* time until initial increase in temperature from beginning of cold water immersion, *T*_*max*_ maximum temperature reached during cold water immersion, *D*_*max*_ time to appearance of *T*_max_, *ΔT* amplitude of temperature reaction (*T*_max −_ *T*_min_), *T*_*mean*_ averaged temperature during whole period of water immersion, *Frequency* number of times of CIVD appearances, *N.S.* not significant

### Skin temperatures

**Fig. 1:**
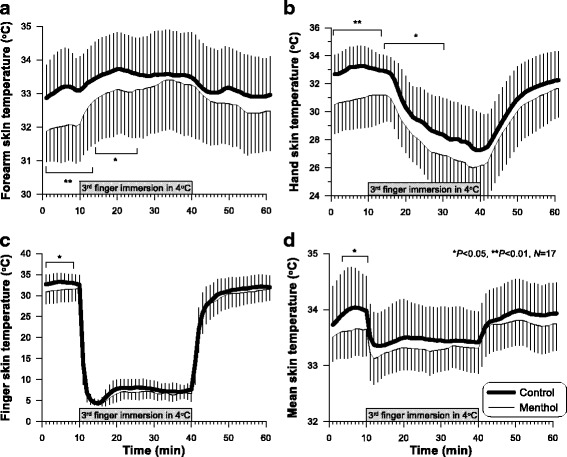
Time courses of forearm skin temperature (**a**), hand skin temperature (**b**), middle finger skin temperature (**c**), and mean skin temperature (**d**) (*N* = 17). Experimental group (menthol) was instrumented each hand and forearm with 1.5% menthol solution 20 min prior to the commencement of the tests. Data were expressed as mean ± SD

The middle finger, hand, forearm and mean skin temperature were significantly influenced by middle finger immersion (*P* < 0.001). Although statistical differences in both conditions were presented only in the hand skin temperatures (*P* < 0.05), not in the finger, forearm, and mean skin temperatures when the data acquired in the entire protocol were computed by the repeated measures ANOVA, significant differences were found in some parts (Table 2). At first, in the initial rest period, the middle finger, hand, forearm, and mean skin temperatures in the menthol condition were significantly lower than those in the control condition (*P* < 0.05; Table 2, Fig. 1). However, after water immersion of the middle finger, the magnitude of differences in forearm and hand skin temperatures between the two conditions continuously reduced and finally disappeared at the 25th min in forearm temperature and 30th min in hand temperature (Fig. 1). An interesting finding was that forearm temperature increased during the 30-min finger immersion for both conditions and the degree of increase was greater in the menthol condition (1.34 ± 0.84 °C) than in the control condition (0.37 ± 1.10 °C) (*P* < 0.001; Table 2). Forearm temperature in the control condition was slightly lower during the recovery than at rest, whereas forearm temperature in the menthol condition was higher in the recovery than at rest (*P* < 0.001; Fig. 1).

**Table 2:** Skin temperatures in a controlled condition and an experimental condition (menthol)

		Control		Menthol		*P* value
		Mean	SD	Mean	SD	
Rest (last 5 min)	*T*_finger_	32.93	2.03	31.55	2.92	N.S.
	*T*_hand_	33.06	1.30	31.22	2.06	0.001
	*T*_forearm_	33.16	1.03	32.16	0.97	< 0.001
	Mean *T*_sk_	34.01	0.66	33.61	0.45	0.012
Immersion (last 3 min)	*T*_finger_	9.01	2.07	8.24	1.90	N.S.
	*T*_hand_	27.31	2.64	26.10	2.38	N.S.
	*T*_forearm_	33.48	1.32	33.24	1.53	N.S.
	Mean *T*_sk_	33.45	0.55	33.35	0.45	N.S.
Recovery (last 3 min)	*T*_finger_	32.10	2.85	31.30	2.75	N.S.
	*T*_hand_	32.15	2.11	31.63	2.12	N.S.
	*T*_forearm_	32.91	1.15	32.63	1.09	N.S.
	Mean *T*_sk_	33.92	0.55	33.72	0.49	N.S.
Change in immersion (*T*_immersion_ *− T*_rest_)	*T*_finger_	− 25.65	2.47	− 24.77	2.73	N.S.
	*T*_hand_	− 5.96	2.76	− 4.65	2.46	N.S.
	*T*_forearm_	0.37	1.10	1.34	0.84	< 0.001
	Mean *T*_sk_	− 0.59	0.37	− 0.33	0.28	0.012
Change in recovery (*T*_rec_ *− T*_rest_)	*T*_finger_	− 0.82	1.65	− 0.25	2.51	N.S.
	*T*_hand_	− 6.14	3.15	− 5.14	2.44	N.S.
	*T*_forearm_	− 0.39	0.58	0.22	0.24	< 0.001
	Mean *T*_sk_	− 0.99	0.33	0.11	0.34	0.021

*N* = 17 females. Subjects’ left hand and forearm were applied with 1.5% menthol solution, and the middle finger of the dominant hand was immersed in 4 °C water

*N.S.* not significant

### Cold pain sensation and thermal sensation

**Fig. 2:**
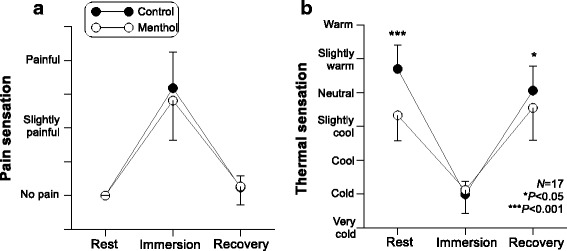
Cold pain sensation (**a**) and thermal sensation (**b**) with corresponding finger temperature. Averaged value of all ratings within identical phase was represented. Experimental group (menthol) was instrumented each hand and forearm with 1.5% menthol solution (*N* = 17). Data were expressed as mean ± SD

Both cold pain sensation and thermal sensation were significantly influenced by phases (*P* < 0.001). Cold pain sensation induced by the local cold test did not show any difference between the two conditions (*P* = 0.464). In contrast to cold pain sensation, there was a significant group difference in thermal sensation (*P* = 0.003). In particular, menthol induced significant differences in thermal sensation at rest and during recovery, and subjects expressed as being cooler for the menthol condition than for the control (Fig. 2; *P* < 0.05). The difference between the two conditions was smaller during recovery than during initial stabilization. Interestingly, thermal sensation during cold water immersion was not affected by menthol application.

## Discussion

While immersing the finger into cold water stimulates cold receptors along with causing heat loss from the skin through conduction, applying menthol solution on the skin only stimulates cold receptors with the minimum conductive heat loss. In the present study, the menthol condition caused more stimulation of cold receptors with the identical conductive heat loss as the control condition. According to our findings, stimulating more cutaneous cold receptors without additional conductive skin cooling did not induce a reduction in the occurrence of CIVD nor significant delay of the first CIVD, but intensified overall vasoconstriction throughout the cold-water immersion after the first occurrence of CIVD, which was represented as the lowered *T*_max_ and *T*_mean_ in the present study. These findings imply stimulating greater number of cold receptors in the adjacent skin without conductive heat loss could not advance triggering of CIVD, but play a role to maintain sympathetic vasoconstrictor tone. These findings may imply that triggering CIVD responses at a particular *T*_min_ is driven by a somewhat different mechanism from that of sustained vasoconstriction during the 30-min cold water immersion. A possible explanation for the indistinguishable onset time between the two conditions is that conductive heat loss through the middle finger and its adjacent skin may play a greater role in triggering the first CIVD along with activation of cold receptors, which is supported by Sendowski et al. [8]. Sendowski et al. [8] demonstrated delayed onset time when the entire right hand along with an index finger was immersed in cold water (T2) than when only the finger was immersed (T1). However, the onset time was not different when the other hand (left hand) along with the right index finger was immersed (T3), although the concentrations of plasma NE in T3 was elevated as much as the T2. From their findings, they concluded that the onset of CIVD could be affected by local cooling on the adjacent skin, independently of the general sympathetic activation.

There are relatively few recent studies supporting locally driven mechanism of CIVD when compared to those supporting the involvement of the central sympathetic nervous system [6]. Daanen [1] summarized the potent underlying mechanisms of CIVD which have been reported in the previous studies into four categories including axon reflex, changes in neurotransmitter, release of dilating substances, and direct influence on vascular smooth muscle. Among these, the third and last are the most important explanatory factors for a locally driven mechanism. Meanwhile, with regard to the release of dilating substances, there is evidence of a link between CIVD and nitric oxide (NO) concentration in birds [18]. This is also supported by a human study on the involvement of NO in the cutaneous vasoconstrictor response to local cooling [19], though the results of these studies cannot be precisely applied into the explanation of CIVD. Johnson and Kellogg [9] briefly stated that the latent vasodilation might be a phenomenon of smooth muscle energetics. Triggering CIVD was not influenced by the amount of cold receptors stimulated only. *T*_max_ is independent of *T*_min_ during initial cooling, but is related and clearly influenced by adding the chemical stimulation of cutaneous cold receptors under the limited conductive heat loss. Nevertheless, the present results implied another underlying mechanism which along with the sympathetic stimulation could contribute to the occurrence of CIVD. For this reason, we could carefully venture the suggestion that the initial process during the CIVD test could be operating by a local mechanism.

However, influence of the central nervous system on the onset time is still in dispute because previous studies strongly presented evidence for that increased core and mean body temperature could influence the occurrence of CIVD even though the cold exposure surrounding cooling body region was controlled [6]. In particular, Flouris and his colleagues [6] pointed out that finger temperatures when CIVD was observed varied from 7.2 to 33.5 °C throughout the whole experimental trials, which could suggest evidence of a weak relationship between local skin temperature and the control of CIVD. However, there may be two potent reasons for this disagreement. Firstly, the initial onset time could be distinguished because the underlying mechanisms of initial vasoconstriction and sustained vasoconstriction would not exactly correspond with each other [9], though the effect of dilating substances seems to be located in the latter process. Johnson and Kellogg [9] also documented unrevealed latent vasodilator mechanisms and carefully suggested the possibility of smooth muscle energetics, not adrenergic or sensory involvement. Nonetheless, this is in line with the present data, which supported distinction between the magnitude and onset time of CIVD. Indeed, the magnitude of CIVD could be related to sustained vasoconstriction rather than the initial vasoconstriction. Secondly, the relative contribution of mean skin temperature and core temperature could be considerably different in a centrally operating system of CIVD [5]. Reduction of 0.5 °C in mean skin temperature or additional signal of cold exposure on the forearm and hand with a decrease of 1 and 2 °C respectively on the regional skin temperature did affect *T*_max_ and *T*_mean_ but not the onset time. To sum up the present data and aforementioned studies at least, initial onset time of CIVD did not change with a 1 and 2 °C decrease in hand and forearm temperature respectively, which could imply the contribution of type of additional local cooling to CIVD onset time with assumption that there is no difference in core body temperature which is likely one of the most influential factors for CIVD.

As much as the mark on the onset time, it deserves to be explained more that simply activating cutaneous cold receptors by the menthol application with the decrease of 1 °C in regional skin temperature significantly enhanced peripheral vasoconstriction throughout cold-water immersion after the first vasodilation. These results could be useful when considering that menthol is a wide-spread ingredient in a lot of household products. Thanks to its cooling effect on the skin, it is widely used in after sun creams, lotions, and pain killers. Likewise, so far, research on the effect of intervention of l-menthol on the thermoregulatory system tends to be concentrated on heat stressed conditions due to its perceptual cooling effect [20, 21]. However, menthol is an agonist of the TRPM8 channel, which serves as a cutaneous thermosensor activating several cold-defense responses [14, 22]. 1.5% menthol induced only a slightly cool sensation (Fig. 2), but resulted in generally enhanced vasoconstriction.

On the other hand, one of the unavoidable questions regarding the application of l-menthol is whether the amount of thermoreceptors stimulated was appropriate enough to compare CIVD responses. The physiological and psychological effects of menthol depend on its idiosyncratic effects [23], the temporal duration and interval [24, 25] and the dose applied on the skin [26]. Differing instrumentation for menthol application could induce various effects perceptually from comfortable coolness to burning pain and from vasoconstriction to inflammation vasodilation in a thermoregulatory aspect. Previous studies where researchers used menthol as attenuating substances of perceptual heat strain tended to use l-menthol in concentration of 0.8% [20]. 0.05% menthol induced cool sensations [27], and 0.2% menthol enhances not only coolness but heat storage [21]. The 1.5% concentration of this study is thought to be an adequate concentration to arouse thermoregulatory changes without pronounced irritation when it is applied only to the forearm.

## Conclusions

A 1.5% menthol application on the forearm and hand enhanced vasoconstriction after the first vasodilation during the cold-water immersion of the finger, but it did not significantly affect the time of initial occurrence of CIVD nor the frequencies of CIVD. From the results, it was concluded that stronger stimulation on cold receptors through the menthol application without additional conductive skin cooling could intensify cutaneous vasoconstriction. However, the present findings did not demonstrate that the trigger of CIVD was also influenced by the stimulation on cold receptors. The present findings implied the contribution of central sympathetic vasoconstrictor activity is relatively greater in retaining vasoconstriction during cold-water immersion than in the first occurrence of CIVD. It was carefully suggested that the possibility of the differentiated underlying mechanisms in initial vasoconstriction strongly related to triggering CIVD and the magnitude of sustained vasoconstriction and that the first occurrence of CIVD could be relatively largely contributed by a locally operated vasodilation mechanism rather than central sympathetic stimulation.
